# Development and validation of a nomogram based on immune-inflammation-nutrition indictors for predicting 28-day mortality in sepsis patients with severe fungal pneumonia

**DOI:** 10.3389/fcimb.2026.1820628

**Published:** 2026-05-05

**Authors:** Gongliang Du, Longyang Ma, Xingbo Dang, Wei Dai, Qi Xin

**Affiliations:** Department of Emergency Surgery, Shaanxi Provincial People’s Hospital, Xi’an, China

**Keywords:** fungal pneumonia, immune-inflammation-nutrition indicators, mortality, nomogram, sepsis

## Abstract

**Background:**

Severe fungal pneumonia with sepsis carries high mortality. Early and accurate prognosis is essential for improving outcomes. We developed and validated a nomogram based on immune-inflammation-nutrition indicators to predict 28-day mortality in these patients.

**Methods:**

We conducted a retrospective, exploratory cohort study analyzing 486 sepsis patients with severe fungal pneumonia, randomly splitting them into training (n=365) and validation (n=121) sets. Using LASSO regression and multivariate logistic regression, we identified independent predictors from clinical, laboratory, and immune-inflammation-nutrition data. We built a nomogram and evaluated its discrimination, calibration, and clinical utility with ROC curves, calibration plots, and decision curve analysis (DCA).

**Results:**

Eight independent predictors entered the nomogram: respiratory failure (RF), chronic obstructive pulmonary disease (COPD), prothrombin time (PT), glucose, blood urea nitrogen (BUN), white blood cell count (WBC), albumin-to-alkaline phosphatase ratio (AAPR), and lactate-albumin ratio (LAR). The nomogram achieved AUCs of 0.884 (training) and 0.834 (validation), outperforming the SOFA score (0.740 and 0.691). Furthermore, temporal validation using an independent later cohort achieved an AUC of 0.875. Calibration curves showed good agreement between predicted and observed outcomes. DCA confirmed clinical utility across a wide range of threshold probabilities.

**Conclusion:**

We developed and internally validated a practical nomogram that integrates clinical variables with immune-inflammation-nutrition indicators to predict 28-day mortality in sepsis patients with severe fungal pneumonia. This tool may help clinicians with early risk stratification and individualized treatment decisions.

## Introduction

Sepsis is a leading cause of death in intensive care units (ICUs) worldwide (Singer et al., 2016). Secondary infections worsen the clinical course, and fungal pneumonia is a particularly serious complication (Pappas et al., 2016). Globally, severe fungal infections represent a growing challenge in critical care. Recent international surveillance data highlight shifting epidemiological patterns, including a rising proportion of non-albicans *Candida* species with reduced antifungal susceptibility. Additionally, the surge in post-viral invasive aspergillosis during respiratory viral pandemics has further underscored the worldwide burden of these infections (Ikuta et al., 2024). Invasive fungal infections (e.g., *Aspergillus* and *Candida* species) are linked to high morbidity and mortality, often exceeding 50%, due to delayed diagnosis, pathogen virulence, and the host’s compromised immunity (Schauwvlieghe et al., 2018; Vazquez et al., 2025). Prognosis in sepsis patients with severe fungal pneumonia depends on the initial septic insult, organ dysfunction, comorbidities, and immunologic and nutritional status (Busani et al., 2020; Garzón Herazo et al., 2020; Kallel et al., 2020; Zhang et al., 2020). The Sequential Organ Failure Assessment (SOFA) score is widely used to predict mortality (Singer et al., 2016), but its accuracy may be limited in specific subgroups such as fungal superinfections because it does not capture the unique pathophysiology or the host immune and metabolic responses (Wang et al., 2023; Sherak et al., 2024).

Biomarkers and composite indices that represent patients’ immune, inflammatory, and nutritional status have drawn more attention in recent years. Neutrophil-to-lymphocyte ratio (NLR), prognostic nutritional index (PNI), and albumin-bilirubin (ALBI) grade are examples of immune-inflammation-nutrition indicators that have demonstrated promising prognostic value in a number of critical illnesses, including sepsis, cancer, and liver disease (Wang et al., 2019; Hou et al., 2021; Kinoshita et al., 2021; Xu et al., 2021; Keskinkilic et al., 2024). They offer a practical, low−cost way to measure nutritional reserve, systemic inflammation, and adaptive immune capacity. However, few studies have used them together to predict outcomes in sepsis patients with severe fungal pneumonia.

Nomograms combine multiple prognostic factors into a user−friendly graphical tool that quantifies individual risk (Balachandran et al., 2015). In China, invasive fungal diseases impose a growing public health burden, with shifting pathogen epidemiology (increasing non−*albicans Candida*) and post−pandemic surges in aspergillosis (van de Veerdonk et al., 2025). For this high-risk patient population, we hypothesized that a nomogram included immune-inflammation-nutrition indicators in addition to important clinical variables would offer a better, more individualized prediction of 28-day mortality. In order to accurately predict 28-day mortality in sepsis patients with severe fungal pneumonia, the goal of this study was to create and validate a novel nomogram based on immune-inflammation-nutrition indicators and standard clinical parameters.

## Methods

### Study population

The Institutional Review Board of Shaanxi Provincial People’s Hospital approved this retrospective cohort study (Approval No: 2025R082), which was carried out in compliance with the Declaration of Helsinki. This was a retrospective, single-center, exploratory analysis. The primary objective was to identify potential prognostic factors and to develop a preliminary predictive model.

### Study design

We initially screened consecutive critically ill adult patients (aged ≥ 18 years) diagnosed with sepsis and severe fungal pneumonia who were admitted to the intensive care unit (ICU) of Shaanxi Provincial People’s Hospital from January 2020 to October 2025. To further assess temporal stability, we additionally collected an independent cohort of patients admitted from November 2025 to March 2026 using the same inclusion and exclusion criteria (temporal external validation set).

The diagnosis of sepsis was determined based on the Sepsis-3 criteria (Singer et al., 2016). Severe fungal pneumonia was diagnosed using a combination of microbiological, clinical, radiological, and severity criteria adapted from EORTC/MSGERC and IDSA guidelines, plus the Sepsis−3 framework (Bassetti et al., 2021). All the following components were required: (1) Microbiological evidence: positive fungal culture, microscopy, or histopathology from lower respiratory tract specimens; a quantitative culture threshold ≥10³ CFU/mL in bronchoalveolar lavage fluid to distinguish invasive infection from colonization. (2) Clinical and radiological correlation: acute respiratory symptoms with new or worsening pulmonary infiltrates on chest imaging, independently assessed by at least two intensivists and not attributed to non−fungal causes. (3) Severity criteria: at least one indicator of significant organ dysfunction: (i) respiratory failure requiring mechanical ventilation; (ii) sepsis or septic shock by Sepsis−3 criteria; (iii) hypoxemia (PaO_2_/FiO_2_ <300 mmHg).

The criteria for exclusion were as follows: 1) less than 18 years old; 2) patients who died or were released from the ICU within 24 hours of admission; 3) patients with incomplete medical records or missing important lab data. Of note, prior or ongoing antifungal therapy was not an exclusion criterion; however, detailed data on specific antifungal agents, timing, and duration were not consistently available for all patients and therefore could not be incorporated into the model.

### Handling of potential colonization

We excluded cases with positive fungal cultures but no clinical symptoms or radiographic evidence of active pneumonia. For *Candida* species, we required additional evidence of invasive disease: histopathological confirmation, concurrent candidemia, or clinical progression despite appropriate antibacterial therapy.

### Data collection

We extracted the following from electronic medical records: demographics, vital signs on ICU admission, comorbidities, laboratory results within 24 hours of admission, and clinical interventions. We collected: (1) demographics and vital signs: age, sex, temperature (T), heart rate (HR), respiratory rate (RR), mean arterial pressure (MAP). (2) Comorbidities: hypertension, diabetes, respiratory failure (RF), chronic obstructive pulmonary disease (COPD), acute−on−chronic liver failure (ACFL), cirrhosis, chronic kidney disease (CKD). (3) Severity: SOFA score at admission. APACHE II (Acute Physiology and Chronic Health Evaluation II) scores were calculated for each patient based on the worst physiological parameters within the first 24 hours of ICU admission. (4) Laboratory parameters: coagulation profiles (PT, APTT, INR, PTA, D−dimer, FDP, FIB); liver and kidney function (AST, ALT, albumin, bilirubin, BUN, creatinine, cystatin−C); complete blood count (WBC, neutrophil, lymphocyte, platelet, hemoglobin, RBC, RDW−SD, RDW−CV, MPV); inflammation/nutrition markers (PCT, CRP, glucose, TC, UA). (5) Treatments: continuous renal replacement therapy (CRRT), mechanical ventilation. (6) Outcome: 28−day all−cause mortality after ICU admission. We chose ICU admission as the time zero for 28−day mortality because all patients in our cohort met the diagnostic criteria for severe fungal pneumonia within the first 3 days of admission. Therefore, the 28−day mortality from ICU admission closely approximates the 28−day mortality from diagnosis, minimizing guarantee−time bias. Moreover, ICU admission represents a clinically meaningful and easily accessible starting point for risk stratification in critically ill patients.

### Laboratory quality control

All laboratory tests were performed in the central clinical laboratory of Shaanxi Provincial People’s Hospital, which is accredited by the China National Accreditation Service for Conformity Assessment (CNAS) in accordance with ISO 15189 standards. Internal quality control procedures were performed daily using commercially available control materials at two or three concentration levels, depending on the analyte. The laboratory participates regularly in external quality assessment schemes organized by the National Center for Clinical Laboratories (NCCL) of China. For coagulation parameters (PT, APTT, INR), samples were collected in 3.2% sodium citrate tubes and processed within 4 hours of collection. For biochemical and hematological analyses, samples were processed within 2 hours of collection using automated analyzers (Beckman Coulter AU5800 for biochemistry, Sysmex XN-9000 for hematology) with routine calibration and maintenance according to manufacturer specifications. All test results were reviewed and validated by certified clinical laboratory technicians before release.

### Immune-inflammation-nutrition indicators

We calculated: NLR = neutrophil/lymphocyte; PLR = platelet/lymphocyte; LMR = lymphocyte/monocyte; SII = (neutrophil × platelet)/lymphocyte; SIRI = (neutrophil × monocyte)/lymphocyte; PNI = albumin (g/L) + 5 × total lymphocyte count (×10^9^/L); AAPR = albumin (g/L)/alkaline phosphatase (U/L); ALBI grade = (log_10_ bilirubin [μmol/L] × 0.66) + (albumin [g/L] × –0.085), categorized as per Johnson et al (van de Veerdonk et al., 2025); LAR = lactate (mmol/L)/albumin (g/L).

### Sample size consideration

Based on the rule of thumb for logistic regression requiring at least 10 events per predictor variable (EPV), our final model included eight independent predictors, necessitating a minimum of 80 outcome events. The training set contained 128 deaths (35.1%), yielding an EPV of 16, which exceeds the recommended threshold and supports reliable model development. The validation set (n = 121, 44 deaths) provided adequate sample size for internal validation.

### Statistical analysis

We split the cohort randomly into training (75%) and validation (25%) sets. To evaluate the model’s performance over time and reduce concerns about overfitting, we performed an temporal validation using the independently collected cohort. Continuous variables are presented as median (IQR) and compared by Mann−Whitney U test; categorical variables as n (%) and compared by chi−square or Fisher’s exact test. We performed univariate logistic regression in the training set. Variables with P < 0.05 entered LASSO regression with 10−fold cross−validation to select features. Non−zero coefficients from LASSO were used in multivariate logistic regression to identify independent predictors. We built a nomogram and evaluated discrimination (AUC), calibration (calibration curves), and clinical utility (DCA). We also assessed individual AUCs for each predictor.

To address potential guarantee−time bias, we performed a sensitivity analysis using the date of severe fungal pneumonia diagnosis as the time zero for 28−day mortality. In addition, we compared baseline characteristics and outcomes between patients diagnosed within the first 7 days of ICU admission (early diagnosis group) and those diagnosed after 7 days (late diagnosis group). The nomogram’s predictive performance was also assessed separately in these two subgroups.

All tests were two−sided; P < 0.05 was considered significant. Analyses used SPSS 26.0 and R 4.2.1 with packages “glmnet”, “rms”, “pROC”, “rmda”.

## Results

### Patient selection and baseline characteristics

**Figure 1:**
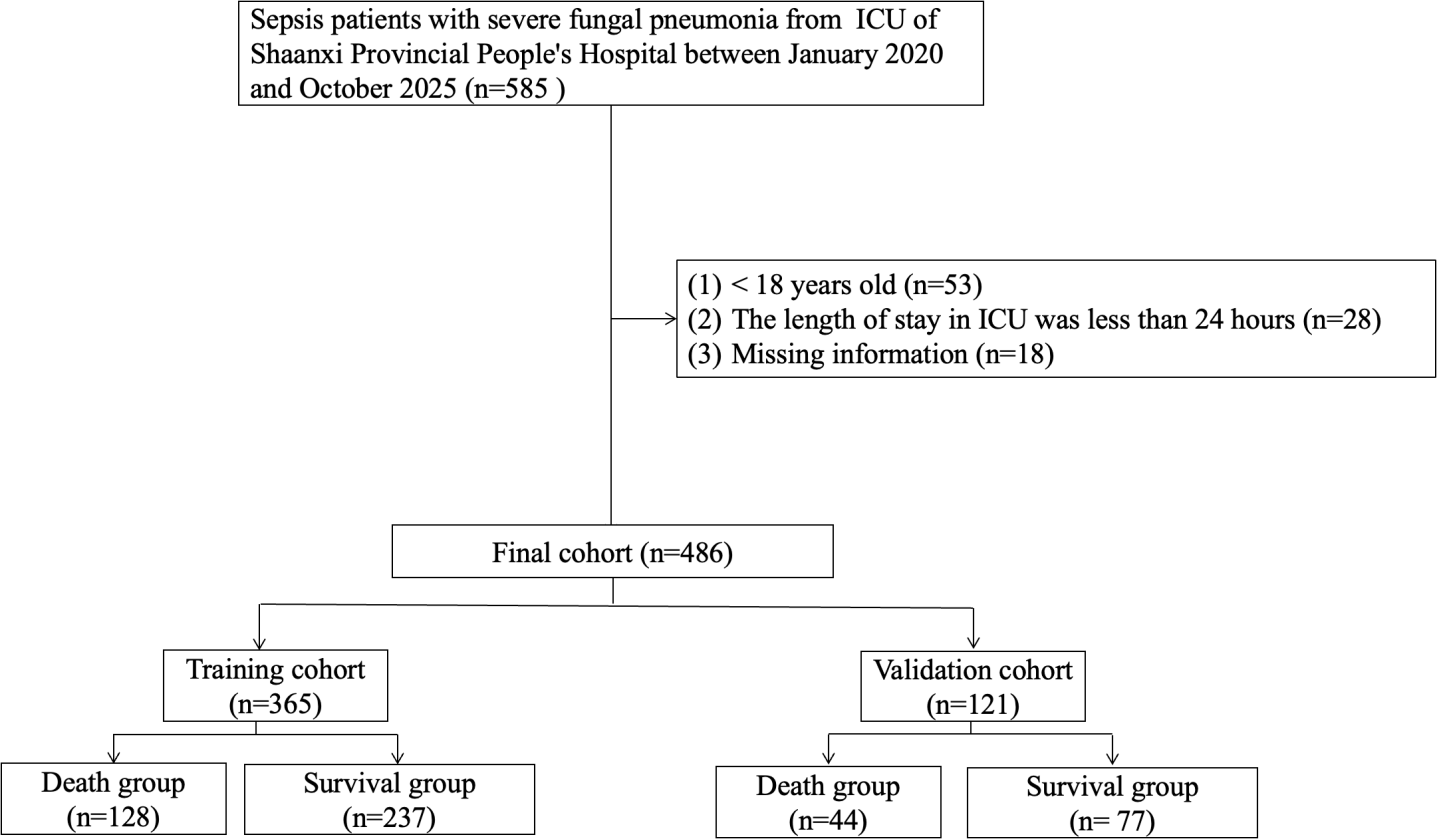
The flowchart of patient selection.

A total of 486 sepsis patients with severe fungal pneumonia were included in this study (**Figure 1**). The cohort was randomly divided into a training set (n = 365) and a validation set (n = 121). Baseline characteristics were balanced between sets (all *P* > 0.05, **Table 1**).

**Table 1:** Baseline characteristics of sepsis patients with severe fungal pneumonia in ICU.

Variable	Total (n =486)	Training (n=365)	Validation (n=121)	*P*-value
Mortality, n (%)				0.882
No	314 (64.6)	237 (64.9)	77 (63.6)	
Yes	172 (35.4)	128 (35.1)	44 (36.4)	
Age (years)	63 (51, 73)	63 (51, 74)	63 (53, 73)	0.994
Gender, n (%)				0.867
Female	280 (57.6)	209 (57.3)	71 (58.7)	
Male	206 (42.4)	156 (42.7)	50 (41.3)	
Fungal pathogen, n (%)				
*Candida* species	312 (64.2)	234 (64.1)	78 (64.5)	0.942
*Aspergillus* species	142 (29.2)	107 (29.3)	35 (28.9)	0.936
Other/mixed	32 (6.6)	24 (6.6)	8 (6.6)	0.998
Vital signs				
T(°C)	36.5 (36.3, 37.2)	36.5 (36.3, 37.2)	36.5 (36.3, 37.2)	0.872
RR (bpm)	21 (19, 25)	21 (19, 26)	21 (19, 24)	0.978
HR (bpm)	98 (86, 109)	97 (86, 109)	98 (85, 109)	0.871
MAP (mmHg)	89.7 (79.3, 100.0)	89.7 (79.0, 100.5)	89.7 (80.3, 99.7)	0.691
Comorbidities				
Hypertension, n (%)				0.795
No	324(66.7)	245 (67.1)	79 (65.3)	
Yes	162(33.3)	120 (32.9)	42 (34.7)	
Diabetes, n (%)				0.978
No	355(73.0)	266 (72.9)	89 (73.6)	
Yes	131(27.0)	99 (27.1)	32 (26.4)	
RF, n (%)				0.978
No	384(79.0)	289 (79.2)	95 (78.5)	
Yes	102(21.0)	76 (20.8)	26 (21.5)	
COPD, n (%)				1
No	437 (89.9)	328 (89.9)	109 (90.1)	
Yes	49 (10.1)	37 (10.1)	12 (9.9)	
ACFL, n (%)				0.799
No	441 (90.7)	330 (90.4)	111 (91.7)	
Yes	45 (9.3)	35 (9.6)	10 (8.3)	
Cirrhosis, n (%)				0.292
No	436 (89.7)	331 (90.7)	105 (86.8)	
Yes	50 (10.3)	34 (9.3)	16 (13.2)	
Septic shock, n (%)				0.927
No	389 (80.0)	293 (80.3)	96 (79.3)	
Yes	97 (20.0)	72 (19.7)	25 (20.7)	
AKI, n (%)				0.669
No	394 (81.1)	298 (81.6)	96 (79.3)	
Yes	92 (18.9)	67 (18.4)	25 (20.7)	
ARDS, n (%)				0.982
No	408 (84.0)	307 (84.1)	101 (83.5)	
Yes	78 (16.0)	58 (15.9)	20 (16.5)	
CKD, n (%)				0.438
No	433 (89.1)	328 (89.9)	105 (86.8)	
Yes	53 (10.9)	37 (10.1)	16 (13.2)	
Laboratory test				
PTA (%)	72.5 (55.0, 87.9)	74.0 (53.9, 88.0)	70.7 (56.5, 84.4)	0.323
TT (s)	17.2 (15.9, 19.1)	17.3 (15.9, 19.0)	17.1 (16.0, 19.2)	0.881
INR	1.29 (1.16, 1.46)	1.28 (1.16, 1.46)	1.30 (1.17, 1.48)	0.440
FDP (mg/L)	11.1 (4.9, 35.3)	11.0 (4.8, 36.2)	12.3 (5.5, 26.0)	0.810
D-D (mg/L)	3.59 (2.18, 8.47)	3.46 (2.14, 8.60)	3.90 (2.18, 7.58)	0.950
FIB (g/L)	4.63 (2.95, 6.21)	4.63 (2.95, 6.18)	4.63 (3.07, 6.32)	0.889
APTT (s)	43.5 (38.7, 49.3)	43.5 (38.7, 49.3)	43.5 (38.9, 49.6)	0.777
PT (s)	15.7 (14.7, 18.1)	15.7 (14.7, 17.6)	15.7 (14.7, 19.0)	0.557
Globulin (g/L)	26.1 (22.5, 30.0)	25.9 (22.5, 29.8)	26.3 (22.7, 30.3)	0.629
AST (U/L)	32.0 (22.0, 62.0)	33.0 (22.0, 67.5)	31.0 (21.5, 59.0)	0.841
ALT (U/L)	27.0 (18.0, 51.0)	26.0 (18.0, 50.5)	30.0 (17.5, 52.0)	0.684
Glucose (mmol/L)	7.20 (5.33, 10.56)	7.23 (5.44, 10.42)	6.79 (5.15, 10.80)	0.855
WBC (× 10^9^/L)	8.27 (4.33, 15.86)	8.13 (4.33, 14.98)	9.00 (4.32, 17.09)	0.434
RDW-SD (fL)	48.2 (44.1, 54.4)	48.0 (44.2, 54.1)	48.3 (44.0, 54.8)	0.814
RDW-CV (%)	14.4 (13.3, 16.0)	14.5 (13.3, 16.0)	14.2 (13.3, 15.9)	0.798
Hemoglobin (g/L)	101 (84, 119)	101 (85, 119)	99 (79, 119)	0.176
RBC (× 10^12^/L)	3.29 (2.80, 3.93)	3.30 (2.83, 3.98)	3.22 (2.72, 3.83)	0.199
MPV (fL)	10.6 (9.6, 11.8)	10.6 (9.6, 11.8)	10.8 (9.6, 12.1)	0.507
PCT (ng/ml)	1.70 (0.35, 11.41)	1.55 (0.31, 13.14)	2.33 (0.52, 9.70)	0.424
CRP (mg/L)	85 (30, 168)	86 (32, 176)	84 (21, 138)	0.449
UA (μmol/L)	295 (186, 411)	296 (186, 416)	293 (181, 406)	0.674
TC (mmol/L)	2.92 (2.05, 3.79)	2.92 (2.05, 3.77)	2.91 (2.06, 3.97)	0.767
Cystatin-C (mg/L)	1.50 (1.07, 2.47)	1.48 (1.04, 2.43)	1.54 (1.17, 2.63)	0.211
BUN (mmol/L)	9.7 (6.0, 16.8)	9.6 (5.6, 16.1)	10.3 (6.4, 19.0)	0.100
Cr (μmol/L)	81.0 (54.8, 169.3)	79.0 (54.0, 166.0)	91.0 (55.0, 208.5)	0.239
CRRT, n (%)				0.104
No	355 (73.0)	274 (75.1)	81 (66.9)	
Yes	131 (27.0)	91 (24.9)	40 (33.1)	
Mechanical ventilation, n (%)				0.285
No	303 (62.3)	233 (63.8)	70 (57.9)	
Yes	183 (37.7)	132 (36.2)	51 (42.1)	
SOFA	7 (6, 10)	7 (6, 9)	8 (7, 10)	0.104
APACHE II	17 (15, 21)	17 (15, 21)	17 (15, 21)	0.465
Hospital LOS (days)	16 (10, 27)	18 (10, 27)	14 (9, 26)	0.106
immune-inflammatory-nutritional indicators				
AAPR (ratio)	0.30 (0.17, 0.44)	0.30 (0.18, 0.46)	0.31 (0.16, 0.42)	0.515
ALBI grade				0.667
grade 1	29 (6.0)	23 (6.3)	6 (5.0)	
grade 2	287 (59.1)	218 (59.7)	69 (57.0)	
grade 3	170 (34.9)	124 (34)	46 (38.0)	
NLR (ratio)	12.0 (5.7, 24.5)	11.7 (5.6, 24.7)	13.3 (5.9, 24.4)	0.860
PLR (ratio)	160 (59, 270)	149 (58, 264)	177 (63, 270)	0.279
LMR (ratio)	1.65 (1.00, 2.87)	1.68 (0.99, 3.00)	1.55 (0.99, 2.77)	0.302
SIRI (ratio)	4.28 (1.64, 10.81)	4.13 (1.61, 10.41)	5.12 (2.01, 14.74)	0.280
PNI (ratio)	32.1 (27.9, 37.3)	32.1 (28.2, 37.7)	32.0 (27.4, 37.0)	0.520
SII (ratio)	1272 (524, 3438)	1238 (534, 3494)	1321 (424, 3147)	0.755
LAR (ratio)	0.06(0.04, 0.10)	0.06 (0.04, 0.10)	0.06 (0.04, 0.11)	0.652

T, body temperature; HR, heart rate; RR, respiratory rate; MAP, mean arterial pressure; ARDS, acute respiratory distress syndrome; RF, respiratory failure; COPD, chronic obstructive pulmonary disease, ACFL, Acute-on-chronic liver failure; CKD, chronic kidney disease; AKI, acute kidney injury; PTA, prothrombin activity; INR, international normalized ratio; PT, prothrombin time; APTT, activated partial thromboplastin time; D-D, D-dimer; FDP, fibrinogen degradation products; FIB, fibrinogen; AST, aspartate transaminase; ALT, alanine aminotransferase; WBC, white blood cell; RDW-CV, red cell distribution width-coefficient of variation; RDW-SD, red cell distribution width-standard deviation; MPV, mean platelet volume; PCT, procalcitonin; CRP, C-reactive protein; UA, uric acid; TC, total cholesterol; CRRT, continuous renal replacement therapy; BUN, blood urea nitrogen; Cr: creatinine; SOFA, sequential organ failure assessment; ICU, intensive care unit; LOS, Length of stay; AAPR, albumin-to-alkaline phosphatase ratio; ALBI, albumin-bilirubin; NLR, neutrophil-lymphocyte ratio; PLR, platelet-to-lymphocyte ratio; LMR, lymphocyte-to-monocyte ratio; SIRI, systemic inflammation response index; PNI, prognostic nutritional index; SII, systemic immune-inflammation index; LAR, lactate-albumin ratio.

### Comparison of early vs. late diagnosis

Of the 486 patients, 412 (84.8%) were diagnosed with severe fungal pneumonia within the first 7 days of ICU admission (early diagnosis group), and 74 (15.2%) were diagnosed after day 7 (late diagnosis group). The 28−day mortality from ICU admission was 34.5% in the early group and 40.5% in the late group (P = 0.312). When using diagnosis date as time zero, the 28−day mortality was 35.1% and 38.9%, respectively (P = 0.547). There was no significant difference in mortality rates between the two groups, indicating that the model is not affected by the time of diagnosis.

### Univariate and multivariate analysis of predictors

Among the 486 enrolled patients, *Candida* species accounted for 312 cases (64.2%), *Aspergillus* species for 142 cases (29.2%), and other/mixed fungal pathogens for 32 cases (6.6%). Univariate logistic regression analysis using *Candida* as the reference group showed that neither *Aspergillus* (OR = 1.336, 95% CI: 0.896-1.992, *P* = 0.154) nor other/mixed pathogens (OR = 0.976, 95% CI: 0.456-2.087, *P* = 0.951) was significantly associated with 28−day mortality.

**Figure 2:**
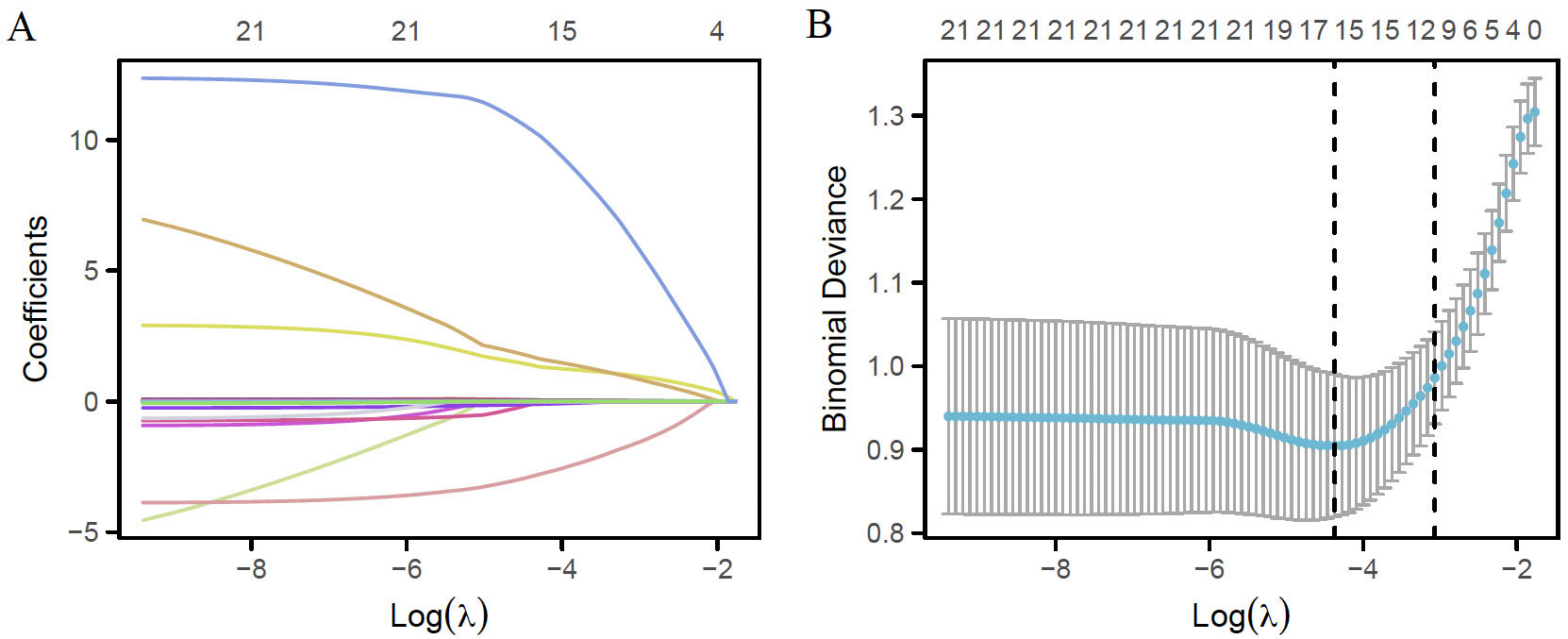
Feature selection via LASSO regression. **(A)** LASSO coefficient profiles of the ten candidate features are displayed, illustrating the trajectory of each coefficient across a sequence of log(lambda) values. At the optimal lambda, twelve features retained non-zero coefficients. **(B)** Parameter tuning for the LASSO model was performed using ten-fold cross-validation. The binomial deviance is plotted as a function of log(lambda), with vertical dashed lines indicating the optimal lambda values selected under the minimum binomial deviance rule and the one standard error (1-SE) criterion.

Univariate logistic regression analysis was performed in the training cohort to identify potential predictors of 28-day mortality (**Table 2**). Variables with a *P* < 0.05 in the univariate analysis were included in the LASSO regression for feature selection. As shown in **Figure 2**, the LASSO model identified twelve non-zero coefficients at the optimal lambda value. Multivariate logistic regression analysis further confirmed eight independent predictors of 28-day mortality: RF, COPD, PT, glucose, BUN, WBC, AAPR, and LAR (**Table 3**).

**Table 2:** Univariate analysis of predictive variables of the 28-day mortality in the training cohort.

Variables	OR	95% CI	*P-*value
Age (years)	0.966	0.625-1.492	0.875
Gender	1.009	0.995-1.022	0.198
Fungal pathogen			
*Candida* species	Reference		
*Aspergillus* species	1.336	0.896-1.992	0.154
Other/mixed	0.976	0.456-2.087	0.951
Vital signs			
T(°C)	0.944	0.796-1.121	0.513
RR (bpm)	1.018	0.986-1.051	0.272
HR (bpm)	1.012	1.001-1.022	0.026
MAP (mmHg)	1.005	0.992-1.018	0.457
Comorbidities			
Hypertension	1.375	0.875-2.162	0.168
Diabetes	1.148	0.710-1.854	0.574
ARDS	4.182	2.321-7.536	< 0.001
COPD	10.058	4.273-23.674	< 0.001
RF	6.072	3.503-10.525	< 0.001
ACFL	6.458	2.921-14.273	< 0.001
Cirrhosis	1.523	0.746-3.111	0.248
Septic shock	3.132	1.844-5.318	< 0.001
AKI	1.317	0.764-2.268	0.322
CKD	1.662	0.837-3.299	0.147
Laboratory test			
PTA (%)	0.991	0.983-1.000	0.039
TT (s)	1.005	0.998-1.012	0.157
INR	1.127	0.761-1.668	0.551
FDP (mg/L)	1.005	0.999-1.011	0.084
D-D (mg/L)	1.046	1.018-1.075	0.001
FIB (g/L)	0.940	0.855-1.034	0.206
APTT (s)	1.024	1.005-1.044	0.014
PT (s)	1.052	1.014-1.091	0.007
Globulin(g/L)	1.006	0.982-1.030	0.647
AST (U/L)	1.001	1.000-1.002	0.194
ALT (U/L)	1.000	1.000-1.001	0.348
Glucose (mmol/L)	1.082	1.031-1.136	0.001
WBC (× 10^9^/L)	1.086	1.053-1.119	< 0.001
RDW-SD (fL)	1.006	0.985-1.027	0.589
RDW-CV (%)	1.019	0.944-1.100	0.624
Hemoglobin (g/L)	1.004	0.996-1.013	0.286
RBC (× 10^12^/L)	1.092	0.861-1.384	0.468
MPV (fL)	1.110	0.973-1.267	0.122
PCT (ng/ml)	1.002	0.995-1.010	0.545
CRP (mg/L)	1.002	1.000-1.005	0.065
UA (μmol/L)	1.001	1.000-1.002	0.139
TC (mmol/L)	0.756	0.626-0.913	0.004
Cystatin-C (mg/L)	1.194	1.042-1.370	0.011
BUN (mmol/L)	1.032	1.010-1.055	0.004
Cr (μmol/L)	1.001	1.000-1.002	0.120
Mechanical ventilation	3.407	2.166-5.357	< 0.001
CRRT, n (%)	1.658	1.020-2.694	0.041
SOFA	1.512	1.357-1.684	< 0.001
APACHE II	1.132	1.077-1.189	< 0.001
Hospital LOS (days)	0.893	0.867-0.921	< 0.001
AAPR (ratio)	0.030	0.006-0.118	< 0.001
ALBI	1.484	1.012-2.177	0.043
NLR (ratio)	1.017	1.008-1.027	< 0.001
PLR (ratio)	1.001	1.000-1.001	0.025
LMR (ratio)	0.985	0.949-1.022	0.414
SIRI (ratio)	1.020	1.007-1.034	0.003
PNI (ratio)	0.967	0.940-0.995	0.022
SII (ratio)	1.000	1.000-1.000	0.931
LAR (ratio)	503562	3750-67607165	< 0.001

T, body temperature; HR, heart rate; RR, respiratory rate; MAP, mean arterial pressure; ARDS, acute respiratory distress syndrome; RF, respiratory failure; COPD, chronic obstructive pulmonary disease, ACFL, Acute-on-chronic liver failure; CKD, chronic kidney disease; AKI, acute kidney injury; PTA, prothrombin activity; INR, international normalized ratio; PT, prothrombin time; APTT, activated partial thromboplastin time; D-D, D-dimer; FDP, fibrinogen degradation products; FIB, fibrinogen; AST, aspartate transaminase; ALT, alanine aminotransferase; WBC, white blood cell; RDW-CV, red cell distribution width-coefficient of variation; RDW-SD, red cell distribution width-standard deviation; MPV, mean platelet volume; PCT, procalcitonin; CRP, C-reactive protein; UA, uric acid; TC, total cholesterol; CRRT, continuous renal replacement therapy; BUN, blood urea nitrogen; Cr, creatinine; SOFA, sequential organ failure assessment; ICU, intensive care unit; LOS, Length of stay; AAPR, albumin-to-alkaline phosphatase ratio; ALBI, albumin-bilirubin; NLR, neutrophil-lymphocyte ratio; PLR, platelet-to-lymphocyte ratio; LMR, lymphocyte-to-monocyte ratio; SIRI, systemic inflammation response index; PNI, prognostic nutritional index; SII, systemic immune-inflammation index; LAR, lactate-albumin ratio.

**Table 3:** Multivariate logistic regression analysis of independent predictors of the 28-day mortality in the training cohort.

Variables	β	SE	Wald	*P*-value	OR (95% Cl)
RF	1.748	0.380	21.113	< 0.001	5.743 (2.725-12.105)
COPD	2.071	0.578	12.850	< 0.001	7.934 (2.557-24.620)
PT (s)	0.057	0.026	5.489	0.029	1.058 (1.006-1.113)
Glucose (mmol/L)	0.074	0.032	9.453	0.019	1.077 (1.012-1.147)
BUN (mmol/L)	0.034	0.015	5.342	0.021	1.034 (1.005-1.065)
WBC (×10^9^/L)	0.071	0.017	16.394	< 0.001	1.073 (1.037-1.111)
AAPR (ratio)	-3.514	0.791	19.729	< 0.001	0.030(0.006-0.140)
LAR (ratio)	14.565	3.132	21.619	< 0.001	2115046 (4560, 980968651)
Constant	0.144	1.369	0.011	0.916	1.155

RF, respiratory failure; COPD, chronic obstructive pulmonary disease; PT, prothrombin time; WBC, white blood cell; BUN, blood urea nitrogen; LAR, lactate-albumin ratio AAPR, albumin-to-alkaline phosphatase ratio.

### Development and performance of the nomogram

**Figure 3:**
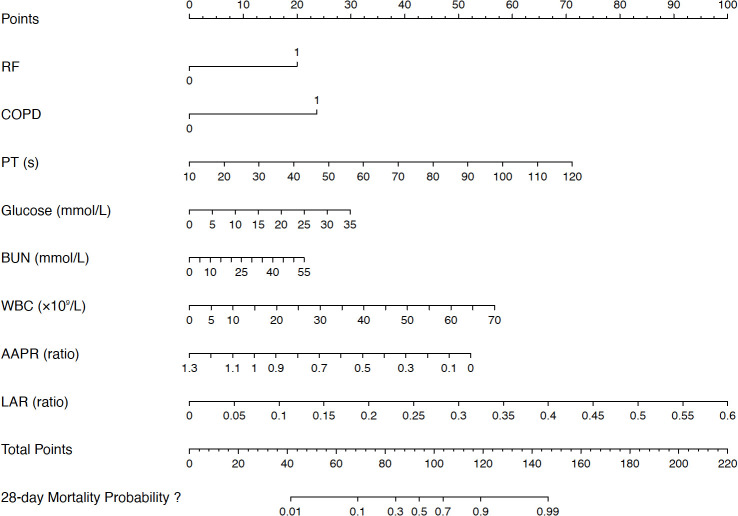
Development of a nomogram to predict 28-day mortality in sepsis patients with severe fungal pneumonia. RF, respiratory failure; COPD, chronic obstructive pulmonary disease; PT, prothrombin time; WBC, white blood cell; BUN, blood urea nitrogen; LAR, lactate-albumin ratio AAPR, albumin-to-alkaline phosphatase ratio. Lower AAPR values correspond to higher points and increased mortality risk.

**Figure 4:**
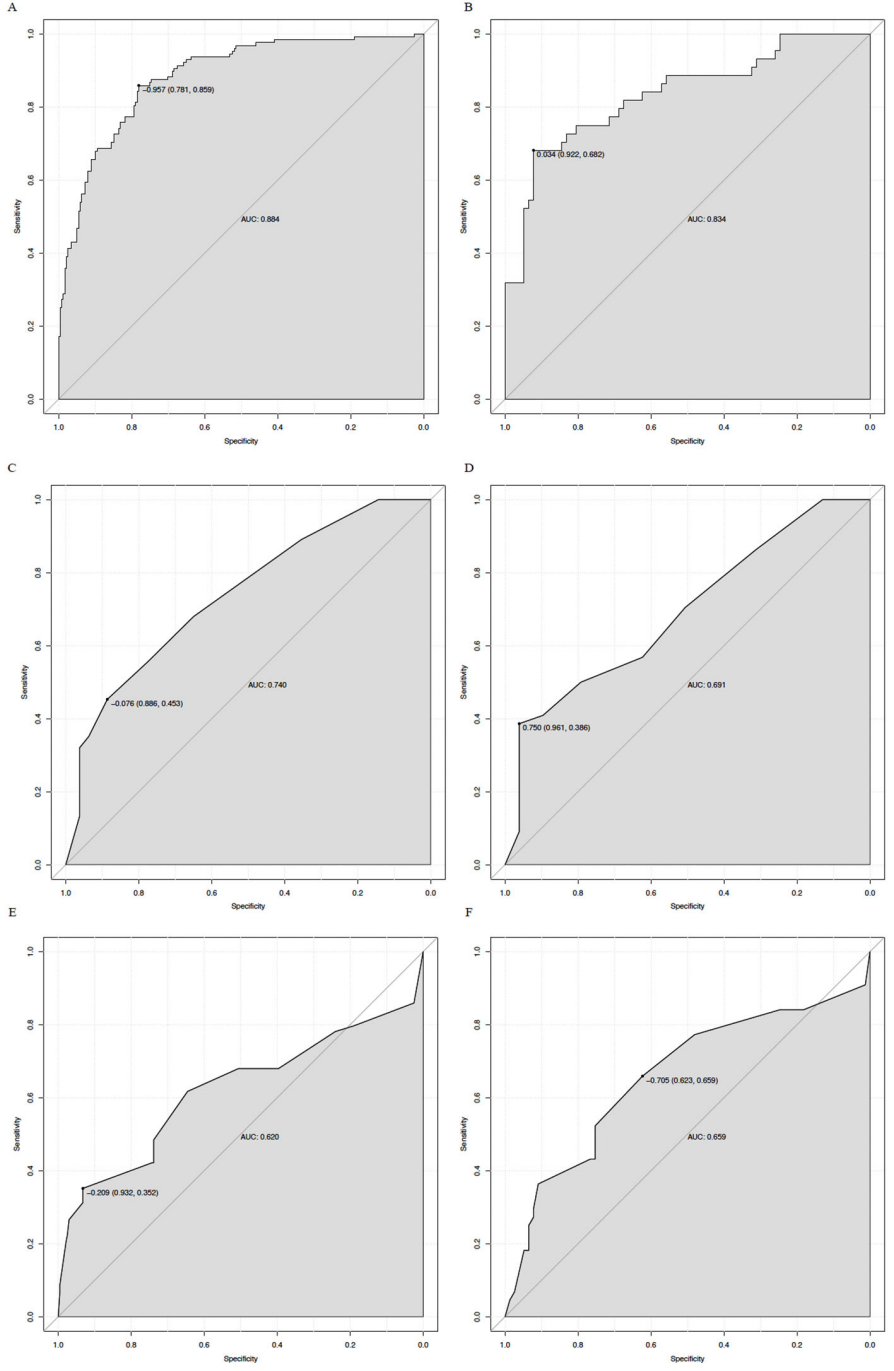
ROC curves of the nomogram and SOFA score for predicting 28-day mortality. The AUC for the nomogram were 0.884 (training set, **(A)**) and 0.834 (validation set, **(B)**); for the SOFA score, the AUCs were 0.740 (training set, **(C)**) and 0.691 (validation set, **(D)**); for the APACHE II, the AUCs were 0.620 (training set, **(C)**) and 0.659 (validation set, **(D)**).

A nomogram was constructed based on the eight independent predictors to estimate the individual probability of 28-day mortality in sepsis patients with severe fungal pneumonia (**Figure 3**). In the training set, the AUC was 0.884, and in the validation set, the AUC was 0.834 (**Figure 4**). These values were superior to those of the SOFA score (training AUC = 0.740, validation AUC = 0.691) and the APACHE II score (training AUC = 0.620, validation AUC = 0.659). The DeLong test showed that the nomogram’s AUC was significantly higher than SOFA in the training set (*P* < 0.001) and validation set (*P* = 0.03). Similarly, the nomogram’s AUC was significantly higher than APACHE II in the training set (*P* < 0.001) and validation set (*P* = 0.009). We further assessed the individual discriminative ability of each independent predictor for 28−day mortality. The AUC in the training set for each variable was as follows: RF 0.652, COPD 0.602, PT 0.677, glucose 0.619, BUN 0.618, WBC 0.710, AAPR 0.711, and LAR 0.705 (**Table 4**).

**Table 4:** The AUCs of independent predictors for 28-day mortality in the training cohort.

Variables	AUC	95%Cl	*P*-value
RF	0.652	0.590-0.715	< 0.001
COPD	0.602	0.539-0.666	0.001
PT (s)	0.677	0.622-0.732	< 0.001
Glucose (mmol/L)	0.619	0.559-0.679	< 0.001
BUN (mmol/L)	0.618	0.559-0.677	< 0.001
WBC (×10^9^/L)	0.710	0.654-0.766	< 0.001
AAPR (ratio)	0.705	0.651-0.760	< 0.001
LAR (ratio)	0.711	0.655-0.766	< 0.001

RF, respiratory failure; COPD, chronic obstructive pulmonary disease; PT, prothrombin time; WBC, white blood cell; BUN, blood urea nitrogen; LAR, lactate-albumin ratio AAPR, albumin-to-alkaline phosphatase ratio.

AUC values for binary variables (RF, COPD) are presented for descriptive purposes only. For binary predictors, the ROC curve consists of a single point, and the AUC should be interpreted with caution.

### Calibration and clinical utility

**Figure 5:**
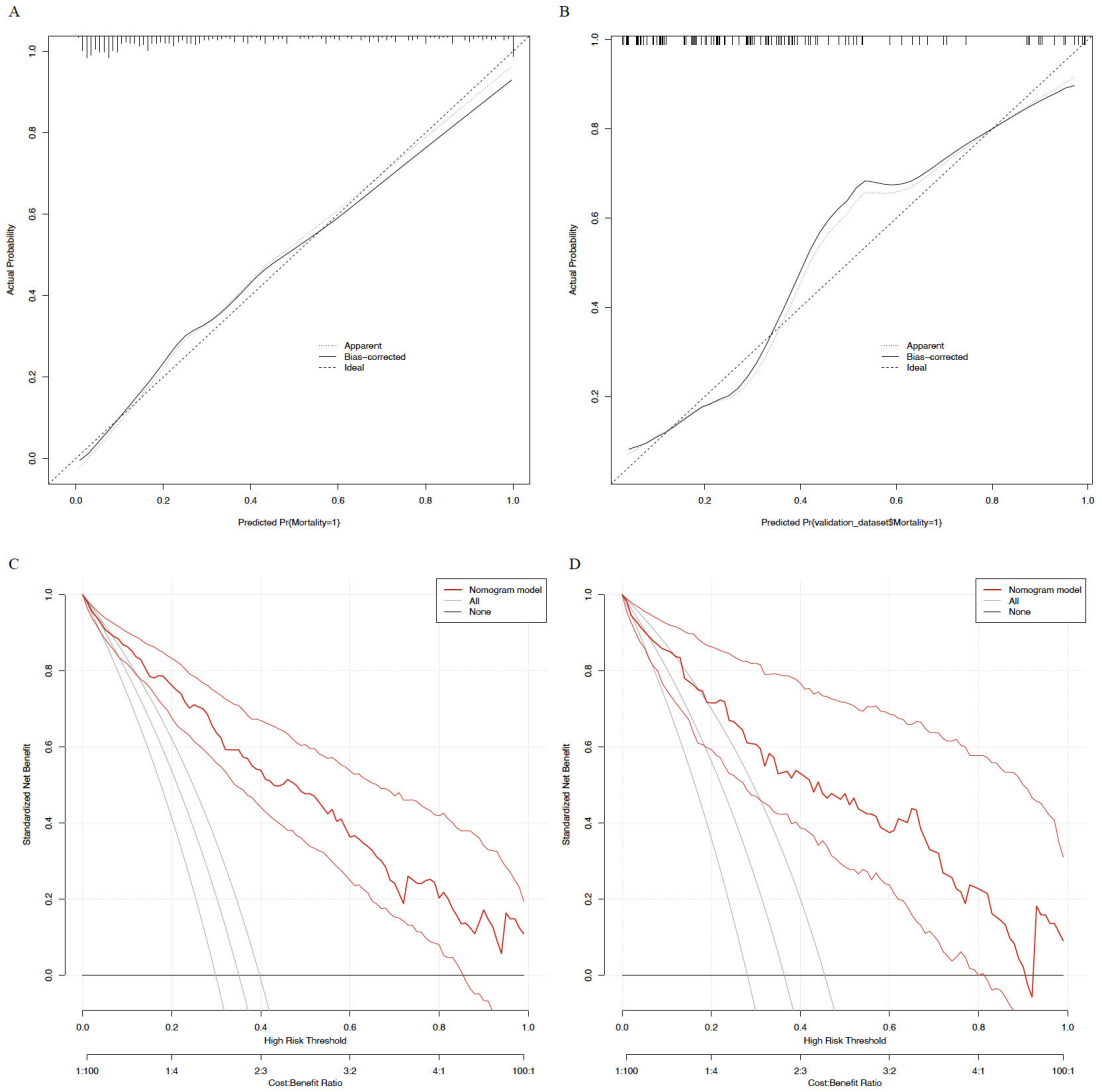
Calibration and decision curve analysis of the nomogram. The calibration curves **(A, B)** depict the agreement between predicted and observed probabilities of 28-day mortality in the training **(A)** and validation **(B)** sets. Decision curve analysis **(C, D)** evaluates the clinical net benefit of the nomogram across different threshold probabilities in the training **(C)** and validation **(D)** sets.

Calibration curves demonstrated good agreement between the nomogram-predicted probabilities and the observed outcomes in both the training and validation sets (**Figure 5A**, 5B). The nomogram’s decision curve remained above both the ‘treat−all’ and ‘treat−none’ lines across a wide range of clinically relevant threshold probabilities (approximately 10% to 100% in the training set and 15% to 90% in the validation set), indicating that the nomogram adds clinical benefit for decision−making when the risk threshold is set within these intervals (**Figures 5C, D**).

### Temporal validation

**Figure 6:**
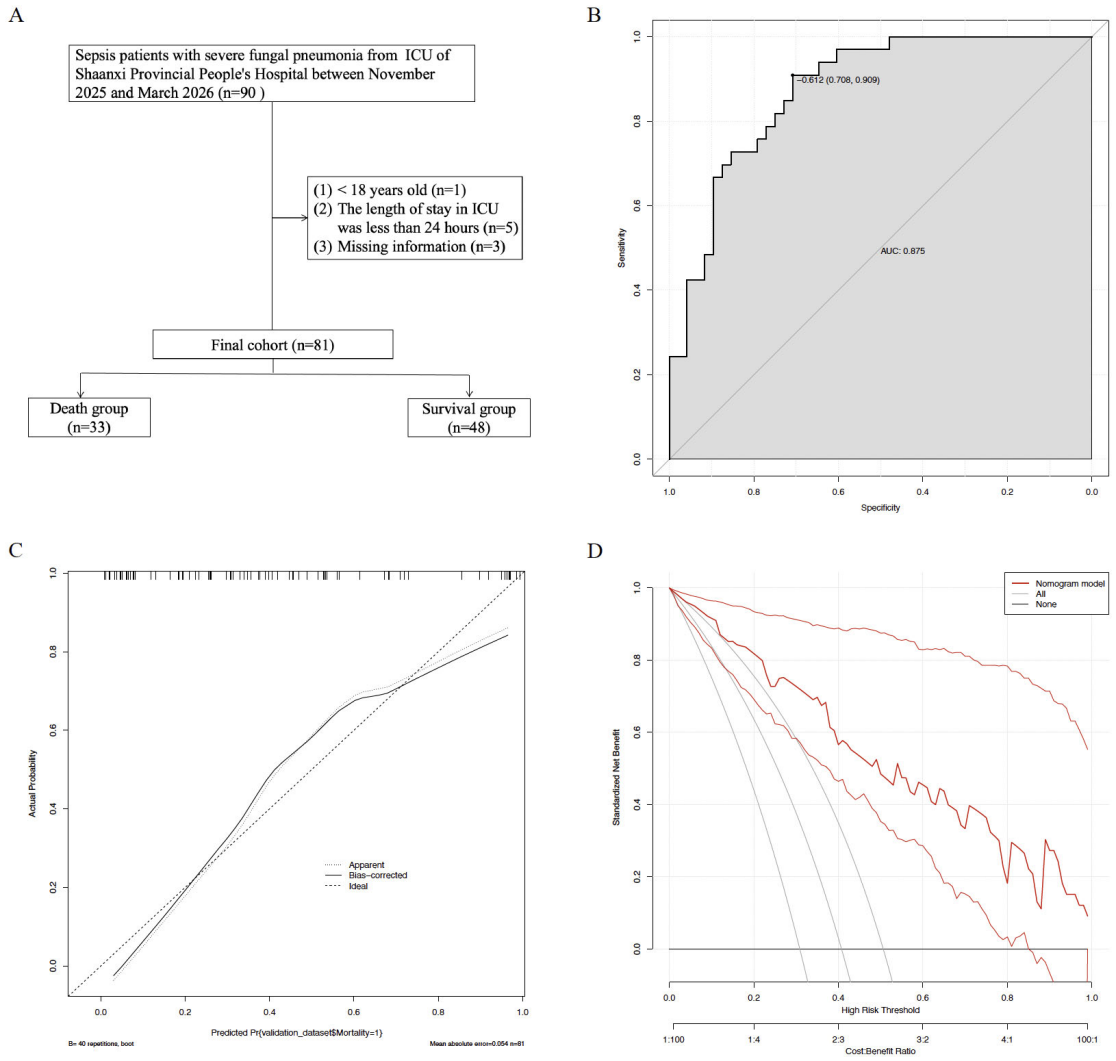
The temporal validation set using the prediction model. The flowchart of patient selection **(A)** from the ICU of Shaanxi Provincial People’s Hospital. The ROC curve **(B)**, Calibration curves **(C)**, and DCA **(D)** of the nomogram for predicting 28−day mortality.

A total of 81 patients were included in the temporal validation set (**Figure 6A**). When applying the original nomogram to this independent cohort, the AUC for predicting 28−day mortality was 0.875 (**Figure 6B**). The calibration curve showed good agreement between predicted and observed risks, and DCA demonstrated a positive net benefit across threshold probabilities of 10%-100% (**Figures 6C, D**). These results confirm the temporal stability and generalizability of our nomogram.

## Discussion

We developed and internally validated a nomogram to predict 28−day mortality in sepsis patients with severe fungal pneumonia. The model integrates eight readily available variables, encompassing comorbidities, routine laboratory parameters, and critically, a panel of composite indicators reflecting the immune, inflammatory, and nutritional status of the host. The nomogram demonstrated superior predictive accuracy compared to the SOFA score and APACHE II. Furthermore, decision curve analysis confirmed its substantial clinical utility, suggesting that the application of this model could facilitate early risk stratification and inform clinical decision-making for this high-risk population. Furthermore, the temporal validation using an independent later cohort yielded an AUC of 0.875, supporting the model’s robustness and temporal stability.

Although *Candida* species are known to frequently colonize the respiratory tract, our stringent inclusion criteria (requiring histopathological confirmation, concurrent candidemia, or clinical progression despite antibacterial therapy) minimized the risk of including colonization as true infection. Among the 486 enrolled patients, *Candida* accounted for 312 cases (64.2%), *Aspergillus* for 142 (29.2%), and other/mixed for 32 (6.6%). Univariate analysis showed no statistically significant difference in 28−day mortality between *Aspergillus* and *Candida* groups (OR = 1.336, *P* = 0.154). This finding suggests that the host response and prognostic factors identified in our nomogram are broadly applicable across different fungal pathogens. From a clinical perspective, while antifungal therapy should be guided by microbiological results and local epidemiology, the mortality risk stratification provided by our nomogram does not require knowledge of the specific fungal species. This simplifies its use in critically ill patients where rapid species identification may be delayed.

First, the presence of pre-existing respiratory comorbidities, namely RF and COPD, were identified as strong predictors of mortality. This finding aligns with previous clinical studies which have consistently shown that underlying chronic lung disease significantly worsens outcomes in patients with severe pneumonia, particularly of fungal etiology (Elbeddini and Tayefehchamani, 2021). For instance, Schauwvlieghe et al. reported that influenza-associated pulmonary aspergillosis (IAPA) carried a mortality rate exceeding 50% in ICU patients, with pre-existing respiratory compromise being a key contributing factor (Schauwvlieghe et al., 2018). The pathophysiological basis for this poor prognosis is multifactorial. RF signifies an acute and often profound deterioration in gas exchange, while COPD reflects a state of chronic lung damage, persistent inflammation, and impaired mucociliary clearance (Agudelo et al., 2020). In the context of fungal pneumonia, these conditions create a critically vulnerable pulmonary environment. The architectural destruction and immune dysfunction in COPD facilitate fungal spore deposition and invasion (Garth and Steele, 2017; Koltsida and Zaoutis, 2021). Subsequently, an exaggerated inflammatory response leads to further alveolar damage and vascular leakage, compounding hypoxemia (Lodge et al., 2022; Adil et al., 2025; Tran et al., 2025). This respiratory compromise not only directly threatens survival but also predisposes to rapid sequential organ failure, as chronic hypoxemia and hypercapnia impair systemic cellular metabolism and resilience (Del Sorbo and Slutsky, 2011; Poon et al., 2015; Tripathi et al., 2018; Alberto Neder and O'Donnell, 2020).

Beyond chronic respiratory conditions, prolonged PT and elevated blood glucose indicate acute coagulation and metabolic disturbances, reflecting severe systemic insult and predicting poor outcomes. Clinical evidence robustly links coagulopathy and hyperglycemia to increased mortality in sepsis and fungal infections (Liu et al., 2022; Ren et al., 2022). A prolonged PT indicates a sepsis-induced coagulopathy, often stemming from systemic inflammatory-mediated endothelial injury, tissue factor expression, and dysregulated thrombin generation, which can progress to microvascular thrombosis and disseminated intravascular coagulation (DIC) (Kim et al., 2020; Maneta et al., 2023; Zhu et al., 2025). This consumptive process impairs organ perfusion and exacerbates ischemic injury. Studies have highlighted that a prothrombotic state was a key feature in patients with septic shock and was associated with multi-organ failure (Levi and Poll, 2015; Semeraro et al., 2015). Concurrently, elevated blood glucose represents a critical metabolic stress response, driven by catecholamine and cortisol release, cytokine-mediated insulin resistance, and enhanced gluconeogenesis. Hyperglycemia is not merely an epiphenomenon; it directly impairs neutrophil function, including chemotaxis and phagocytosis, and disrupts endothelial integrity (Diebel et al., 2019; Farhan et al., 2023). Crucially, in fungal sepsis, elevated glucose can promote fungal growth and virulence, particularly in *Candida* species, by enhancing hyphal formation and biofilm production (Bezerra et al., 2023). Therefore, hyperglycemia was an independent risk factor for invasive candidiasis and subsequent mortality in the ICU, creating a vicious cycle of impaired host defense, exacerbated infection, and amplified inflammation.

The model also underscores the critical prognostic weight of markers indicating renal dysfunction and systemic inflammatory burden, namely elevated BUN and abnormal WBC count. These parameters are integral components of widely used scoring systems like SOFA and APACHE II, and their predictive value is reaffirmed in our specific cohort. An elevated BUN level often signals acute kidney injury (AKI) or prerenal azotemia, reflecting systemic hypoperfusion, direct tubular injury from inflammatory mediators, and an underlying catabolic state. The development of AKI in sepsis is a pivotal event, independently associated with a higer mortality rate, as it signifies the failure of a central homeostatic organ and often necessitates complex fluid and metabolic management (Zarbock et al., 2023). Kallel et al. found that ICU-acquired infections leading to renal dysfunction were a major determinant of fatal outcome (Kallel et al., 2020). Similarly, an elevated WBC count is a direct reflection of a dysregulated and excessive host inflammatory response to severe infection. In the context of sepsis, marked leukocytosis often signifies an overwhelming, yet ineffective, systemic inflammatory burst that can contribute to collateral tissue damage and organ dysfunction (Azevedo et al., 2021). However, within the specific and vulnerable context of fungal sepsis, this apparent hyper-response may paradoxically coincide with or precede a state of functional immune exhaustion. Consequently, sustained leukocytosis serves as a key indicator of lost immunologic homeostasis and is strongly associated with progressive organ failure and poor prognosis in these patients.

Finally, composite indices reflecting nutritional and metabolic reserve offered unique and powerful prognostic insight beyond conventional markers, such as AAPR and the LAR. The integration of these indices addresses the critical interplay between chronic physiological reserve and acute physiological stress. A low AAPR, which combines hypoalbuminemia with elevated alkaline phosphatase, signals both poor nutritional status and potential hepatic involvement or cholestasis. Hypoalbuminemia itself is a well-known negative prognostic factor in critical illness, reflecting chronic malnutrition, systemic inflammation (as a negative acute-phase reactant), and reduced oncotic pressure leading to tissue edema. Wang et al. demonstrated that the ALBI grade, a related liver function index, was an accurate prognostic score in patients with chronic liver disease, and its components are reflected in the AAPR (Zhang et al., 2023). The LAR ingeniously integrates two critical dimensions: lactate as a real-time marker of tissue hypoperfusion and anaerobic metabolism, and albumin as an indicator of chronic nutritional and inflammatory status. A high LAR, therefore, captures the perilous confluence of acute hypoperfusion on a background of chronic catabolism and frailty (Rissel et al., 2022; Xu et al., 2022). This combination identifies a patient subgroup with severely diminished physiological reserve who are least likely to withstand the profound metabolic demands of sepsis and are at the highest risk of mortality, a concept increasingly recognized in the assessment of critically ill patients.

Although each component of our model has been individually linked to sepsis prognosis, their combined application in severe fungal pneumonia represents a novel contribution. The nomogram’s main advantages are: (1) integration of clinical, laboratory, and immune−nutritional indicators into a single tool; (2) specific tailoring to the understudied population of fungal pneumonia in sepsis; (3) reliance on routine, readily available variables; and (4) superior discrimination over the SOFA score. These features make our nomogram a practical and promising tool for early risk stratification in this high−risk setting. Candidemia management faces challenges from diagnostic delays and emerging echinocandin resistance, particularly in *Nakaseomyces glabratus* (Dong et al., 2026). Optimized strategies such as alternating echinocandins with liposomal amphotericin B may reduce resistance (Cai et al., 2026). Our nomogram can complement these approaches by identifying high−risk patients who might benefit from early, targeted antifungal therapy.

### Limitation

Several limitations of this study warrant consideration. First, this is a retrospective, exploratory study conducted at a single center, which inherently limits the generalizability of our findings. Although we performed a temporal validation using an independent later cohort from the same institution, this cohort shares the same institutional practices, patient demographics, and local epidemiology. Therefore, this does not constitute true external validation. Prospective, multi-center external validation in geographically and clinically diverse populations is essential to confirm the model’s robustness and transportability. Second, while we incorporated a comprehensive set of variables, unmeasured confounding factors, such as specific antifungal regimens and their timing, or genetic predispositions, could influence outcomes. Third, the diagnosis of fungal pneumonia, while based on positive microbiology, can be challenging, and some misclassification is possible. Fourth, although we attempted to distinguish colonization from true infection, residual misclassification is possible, especially for *Candida*. Fifth, due to the retrospective single−center design, we lacked exact survival times for all patients and could not perform time−dependent survival analysis (e.g., Cox regression); our model only provides a static 28−day mortality estimate, which is less informative for dynamic clinical decision−making. Sixth, the LASSO and multivariate logistic regression approach assumes a linear relationship between continuous predictors and the log-odds of 28-day mortality. While this assumption facilitates model simplicity and clinical interpretability, it may not fully capture potential non-linear or threshold effects inherent in certain variables (e.g., glucose, BUN). Future studies could explore alternative approaches such as restricted cubic splines to address this limitation. Finally, we did not have complete records of antifungal therapy details (including specific agents, timing, and duration) or precise cumulative doses of glucocorticoids and immunosuppressants. These factors may influence patient outcomes and could not be adjusted for in our model. Future prospective studies should incorporate standardized treatment protocols, collect precise event times and include external cohorts to develop a more robust, time−dependent prognostic model.

## Conclusion

In summary, the nomogram successfully integrates variables representing different yet interconnected physiological domains—chronic organ dysfunction, acute coagulopathy, metabolic stress, renal injury, immune response, and nutritional reserve. This integrated approach provides a more holistic prognosis than traditional scores like SOFA, which focus primarily on acute organ dysfunction. By capturing the multifaceted interaction between the host’s baseline vulnerability and the acute fungal infection, this tool may assist clinicians in early risk stratification and personalized treatment planning for this high-risk population.

## Data Availability

The raw data supporting the conclusions of this article will be made available by the authors, without undue reservation.
